# Are populations of postpartum women differentially served by community health worker programs: an observational cohort study from Zanzibar, Tanzania

**DOI:** 10.1186/s12884-024-06356-8

**Published:** 2024-03-07

**Authors:** Michelle Olakkengil, Samira Said, Omar Abdalla, Rachel Hofmann, Bethany Hedt-Gauthier, Isabel Fulcher

**Affiliations:** 1 Department of Global Health and Population, Harvard T.H. Chan School of Public Health, Boston, USA; 2 D-Tree International, IRCH Building, Kidongo Chekundu, Zanzibar, Tanzania; 3 Department of Global Health and Social Medicine, Harvard Medical School, Boston, USA; 4 Department of Biostatistics, Harvard T.H. Chan School of Public Health, Boston, USA

**Keywords:** Community health worker, Postpartum, Maternal health, Mobile health, Tanzania, Health equity

## Abstract

**Background:**

Although community health worker (CHW) programs focus on improving access to healthcare, some individuals may not receive the intended quality or quantity of an intervention. The objective of this research was to examine if certain populations of pregnant women differentially experience the implementation of a community health worker-led maternal health intervention in Zanzibar.

**Methods:**

We included pregnant women enrolled in the Safer Deliveries (*Uzazi Salama*) program, which operated in 10 of 11 districts in Zanzibar, Tanzania between January 1, 2017, and June 19, 2019 (*N* = 33,914). The outcomes of interest were receipt of the entire postpartum intervention (three CHW visits) and time to first postpartum CHW visit (days). Visits by CHWs were done at the women’s home, however, a telehealth option existed for women who were unable to be reached in-person. We conducted statistical tests to investigate the bivariate associations between our outcomes and each demographic and health characteristic. We used multivariate logistic regression to estimate the relationships between covariates and the outcomes and multivariate linear regression to estimate the association between covariates and the average time until first postpartum visit.

**Results:**

Higher parity (OR = 0.85; *P* = 0.014; 95%CI: 0.75–0.97), unknown or unreported HIV status (OR = 0.64; *p* < 0.001; 95%CI: 0.53–0.78), and receipt of phone consultations (OR = 0.77; *p* < 0.001; 95%CI: 0.69–0.87) were associated with a lower odds of receiving all postpartum visits. Similarly, women with an unknown or unreported HIV status (estimated mean difference of 1.81 days; *p* < 0.001; 95%CI: 1.03–2.59) and those who received a phone consultation (estimated mean difference of 0.83 days; *p* < 0.001; 95%CI: 0.43–1.23), on average, experienced delays to first visit. In addition, current delivery at a referral hospital was associated with lower odds of receiving a postpartum visit and longer time to first visit compared to delivery at home, cottage hospital, PHCU + , or district hospital. Women from all other districts received their first visit earlier than women from Kaskazini B. There were no differences in the odds of receiving the entire postpartum intervention by sociodemographic variables, including age, education, and poverty assessment indicators.

**Conclusion:**

The results indicate no differences in intervention contact across wealth and education levels, suggesting that the program is effectively reaching women regardless of SES. However, women with other characteristics (e.g., higher parity, unknown or unreported HIV status) had lower odds of receiving the complete intervention. Overall, this work generates knowledge on existing disparities in intervention coverage and enables future programs to develop approaches to achieve equity in health care utilization and outcomes.

## Background

The postpartum period, defined by the World Health Organization (WHO) as the 42 days after birth [1], is a particularly vulnerable time for both women and infants. Maternal mortality is highest during this period—in sub-Saharan Africa, 47.8% of all maternal deaths occurred 24 h to 42 days postpartum [2]. Likewise, neonatal mortality (up to 28 days postpartum) accounts for an additional 34.2% of under-5 mortality. Coordinated efforts motivated by both the Millennium Development Goals and the Sustainable Development Goals [3] to decrease maternal and neonatal deaths resulted in a 38% reduction in the maternal mortality rate (MMR) and 40% reduction in neonatal mortality rate worldwide from 2000 and 2017 [4, 5]. Despite these worldwide reductions, Tanzania continues to have high rates of maternal and neonatal mortality [6], with studies indicating that major causes of maternal death were related to preventable postpartum complications [7–9].

Timely and comprehensive postnatal care (PNC) at a health facility has been shown to reduce the risk of maternal and neonatal complications and death [10, 11], in addition to facilitating the provision of services on exclusive breastfeeding, postpartum family planning, immunizations, nutrition, and HIV [12–14]. In resource-limited settings in low- and middle-income countries, the WHO recommends that all women and newborns receive PNC for at least 24 h if the birth occurred at a health facility. If the birth occurred at home, women and newborns should receive their first postnatal contact from midwives, other skilled providers and/or CHWs within 24 h of birth. At least three additional postnatal contacts are recommended following delivery—the first between 48 and 72 h, the second between days 7 and 14, and the third 6 weeks after birth [6]. The WHO also recommends home visits in the first week after birth for care of the mother and newborn. The Tanzania Demographic Health Survey (TDHS) 2015–2016 estimated that only 30.9% of mothers reported seeing a health care personnel (doctor, midwife, nurse, CHW, or traditional birth attendant) within 24 h postpartum with 63% of women reporting no postnatal check-up [15]. In Zanzibar, the location of this study, PNC coverage within the first two days is 40.1%.

In resource-limited settings, health interventions that increase the uptake of PNC visits are critical to improving maternal and neonatal health outcomes. CHW programs that deliver home visits to women and infants during the postpartum period can fill this gap [16–19]. With proper training and support, CHWs have been shown to increase health-seeking behaviors related to exclusive breastfeeding, family planning, and nutrition, and can identify danger signs for both mothers and newborns and support referral for management of complications [1, 20]. The receipt of postnatal care has been linked to lower rates of mortality; for example, a CHW program in Bangladesh found a substantial reduction in neonatal mortality among women who received PNC by trained CHWs within the first two days postpartum [21]. A systematic review evaluating the effect of home visits for PNC also highlighted that neonates who received a home visit within 28 days of birth had 34% lower neonatal mortality than those who received no postnatal visit [22]. Other community-based interventions that provided postpartum home visits managed to identify life-threatening postpartum morbidities (e.g., severe anemia, severe hypertension, secondary postpartum hemorrhage) and reduce rates of postpartum depression and postpartum sepsis in mothers [23–25].

While CHW programs traditionally focus on improving access to health care for vulnerable populations, certain subgroups of women within these programs may not receive the intended quantity or quality of the intervention. As such, it is imperative to understand if CHWs are inadvertently biased against certain groups of women, which in turn could greatly impact which individuals receive and benefit from interventions. Community factors such as disease-related stigma, education status, and knowledge level of the target group have been shown to affect CHW performance [26]. For example, some CHWs perceived people within communities with low levels of education and health knowledge to be “ignorant” and “uncooperative.” [27] One systematic review examined the factors that contributed to the equitability of CHW programs [28] and determined that inequities persisted with those living further from the CHW, as they were less likely to receive household visits [29–31]. Moreover, programs with educational requirements for the CHW resulted in more CHWs being recruited from and operating within communities with higher educational levels, therefore disadvantaging illiterate communities [32]. Overall, the quality of CHW services for different socio-demographic groups and the role CHWs have in addressing social determinants for health is a critical gap in the literature [27]. Differential receipt of postpartum interventions may create lasting health inequities that impact health knowledge, practices, and access to health services, and potentially contribute to maternal and neonatal mortality.

Unfortunately, there is little to no research on how specific CHW programs may differentially serve women during the postpartum period. Research has instead focused on the demographic factors associated with PNC visit attendance at a health facility. In rural Tanzania, parity, wealth index, nearest health facility type, and religion are predictors for women seeking maternal care [33]. Other factors associated with higher PNC visit attendance include lower parity, health facility delivery, urban area of residence, and higher level of education [14, 34]. Even within this literature, few studies have focused on variation in the timing of the first PNC visit by demographic characteristics. If the barriers to attaining high coverage of PNC are not well understood, then such factors will continue to persist in settings where utilization is low, including at the community level [22].

D-tree International and the Zanzibar Ministry of Health designed and implemented the Safer Deliveries program (2016–2019) in 10 of Zanzibar’s 11 districts. The program aimed to reduce maternal and neonatal mortality by increasing rates of facility deliveries and postpartum follow up visits through an integrated community-based digital health system. It is important to note that CHW programs vary by program and country, and in some cases, clients may specifically seek out CHWs in their communities for health services. However, in this particular program, CHWs directly visited and connected pregnant women and their families to existing community resources such as a community transport system and health facilities so that all women have the education, support and resources to deliver in a health facility. The program’s mobile app guided CHWs in providing postpartum home visits to women. The program has since been adopted and expanded as Zanzibar’s national community health program, *Jamii ni Afya* (Community is Health), which reached full national scale in August 2021. The Safer Deliveries program reached nearly 54,000 women and led to an increase in postnatal follow-up care at a facility within 7 days after delivery (35% in 2016 to 93% in 2019), as well as increases in completed postpartum (57.1% in 2016 to 80.6% in 2019) and neonatal referrals (37.5% in 2016 to 93.4% in 2019) [35]. Given the overall success, it’s important to focus in and determine if all women are benefitting or if there are some populations that are inequitably served through the program. This consideration is vital to improving both future programmatic outcomes and overall health equity.

In this paper, we investigate if certain populations of women are *differentially served* by D-tree’s Safer Deliveries program during the postpartum period. To investigate if populations of women were *differentially served* by the program, we investigate potential disparities in the two program outcomes: receiving the full postpartum intervention (three postpartum CHW home visits) and timely receipt of first postpartum CHW home visit. We aim to understand which groups of women did not experience the intended impact of the Safer Deliveries program home visits. This information will help future programs by generating knowledge on intervention coverage and develop targeted approaches to reach specific groups of women and improve overall operations and service delivery.

## Methods

### Safer deliveries program and data collection

The Safer Deliveries (*Uzazi Salama*) program, designed and implemented by D-tree International in collaboration with the Zanzibar Ministry of Health from January 2016 to September 2019, aimed to improve the quality of maternal and neonatal health care through a digital community health volunteer program. Although the program refers to the selected community members as Community Health Volunteers, we will utilize the term CHW in accordance with prior literature.

The program utilized a mobile app developed by D-tree International, built utilizing Logiak (previously referred to as MangoLogic) software. CHWs, with support from the mobile app, enrolled pregnant women in the program and conducted home-based visits to create personalized birth plans based on each woman’s obstetric history and risk factors. The mobile app also assisted CHWs to provide health messages and reminders at the appropriate phase of a woman’s pregnancy, screen for danger signs and coordinate referrals to a health facility, calculate and track savings necessary for transportation and delivery expenses, and link women with a community driver for transportation to a facility for delivery. The data collected by the CHWs on the mobile app were synchronized to the Safer Deliveries server and accessible through program dashboards for real-time monitoring.

CHWs visited women in their homes during their pregnancy and postpartum period. During the postpartum period, CHWs were scheduled to visit mothers and their newborns three times (with additional visits if the woman or newborn was referred for a health danger sign). The three postpartum visits were scheduled to occur within 3 days of delivery (ideally within 24 h), between 3 and 8 days after delivery, and between 8 and 42 days after delivery. During these home visits, CHWs encouraged women to attend postnatal check-ups, screened for postpartum and neonatal danger signs, and continued to provide counseling to ensure healthy practices and outcomes. Notably, some women move away during late months of pregnancy to be closer to their families, affecting the CHW’s ability to follow-up. To account for this, D-tree introduced phone-based visits with abbreviated content in March 2018.

### Study population

**Fig. 1:**
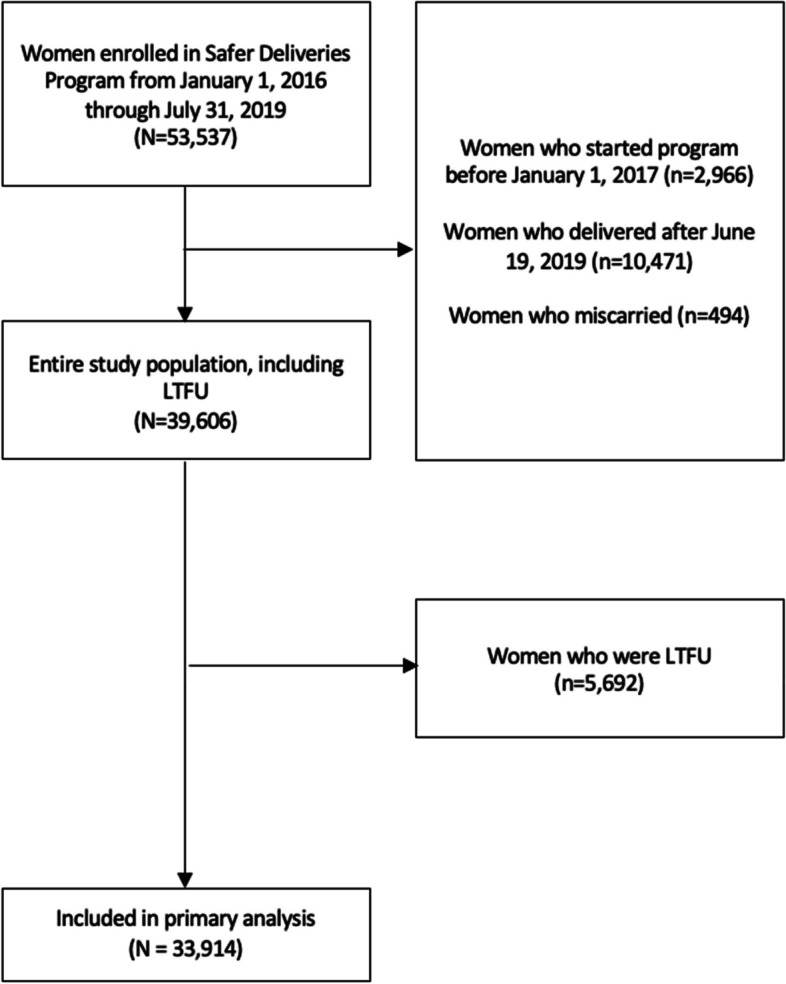
Flow chart of study population

The Safer Deliveries program was implemented on the Pemba and Unguja islands of Zanzibar, a semi-autonomous region of the United Republic of Tanzania. De-identified program data was available for women who enrolled in the program from January 1, 2016 through July 31, 2019 (*N* = 53,537 women). For this study, we considered women who enrolled after one year of program’s initiation, January 1, 2017, to assess the outcomes once the intervention stabilized, and who had a recorded live birth before June 19, 2019 to ensure that participants were eligible to receive the entire postpartum intervention (42-day postpartum period); in total, 39,606 women were captured in this window. There were 5,692 (14.4%) women who were lost to follow-up (LTFU), defined as women enrolled in the program for at least 9 months without a recorded delivery (and any subsequent postpartum visits) by July 19, 2019 or women who did not have a recorded postpartum visit within the 42-day postpartum period. We conducted a separate sub-analysis to investigate the demographic characteristics of women who were LTFU as these women also represent not receiving the intervention as intended (*N* = 5,692). After excluding women LTFU, the final study population used for main analyses was 33,914 women (Fig. 1).

### Variables

#### Outcomes

The two main outcomes of interest were 1) receipt of all postpartum visits, defined as a binary outcome indicating receipt of three visits with the CHW, and 2) time to first postpartum visit, defined as days from delivery to the first visit with the CHW. We considered these outcomes as they characterize the receipt of the full postpartum intervention.

#### Characteristics of enrolled women

We were interested in identifying the sociodemographic and health characteristics that may impact women’s experiences of the intended postpartum intervention. Sociodemographic variables include: maternal age (< 20 years old, 20–29 years old, 30–39 years old, ≥ 40 years old), district of residence (Kaskazini A, Kaskazini B, Kati, Maghribi, Kusini, Mkoani, Wete, Micheweni, Chake Chake), and poverty assessment indicators capturing socioeconomic status (SES): education level (no education, some primary, completed primary, some secondary, completed secondary), electricity access, drinking water access (surface water, tap pump outside, well, tap pump in home, well in home, other), all children currently living, roof material (dirt, plastic mat, concrete, tiles, other), and floor material (scrap corrugated iron, corrugated iron sheets, thatched, tiles/shingles, other). Self-reported maternal health history variables include: HIV status (positive, negative, unknown/unreported), parity (0, 1–2, 3–4, 5–7, 8 + births), previous spontaneous abortion, previous stillbirth, previous and current pregnancy conditions, and previous delivery location (no previous birth, at home/in the community, on the way to a health facility, at health facility). Previous pregnancy condition was defined as the presence of at least one of the following conditions during a previous pregnancy: eclampsia, perineal tear, placenta previa, prolonged labor, retained placenta, postpartum hemorrhage, vacuum, and c-section. Likewise, current pregnancy conditions involved having at least one of the following conditions: twins, breech position, and macrosomia.

##### Programmatic characteristics

**Fig. 2:**
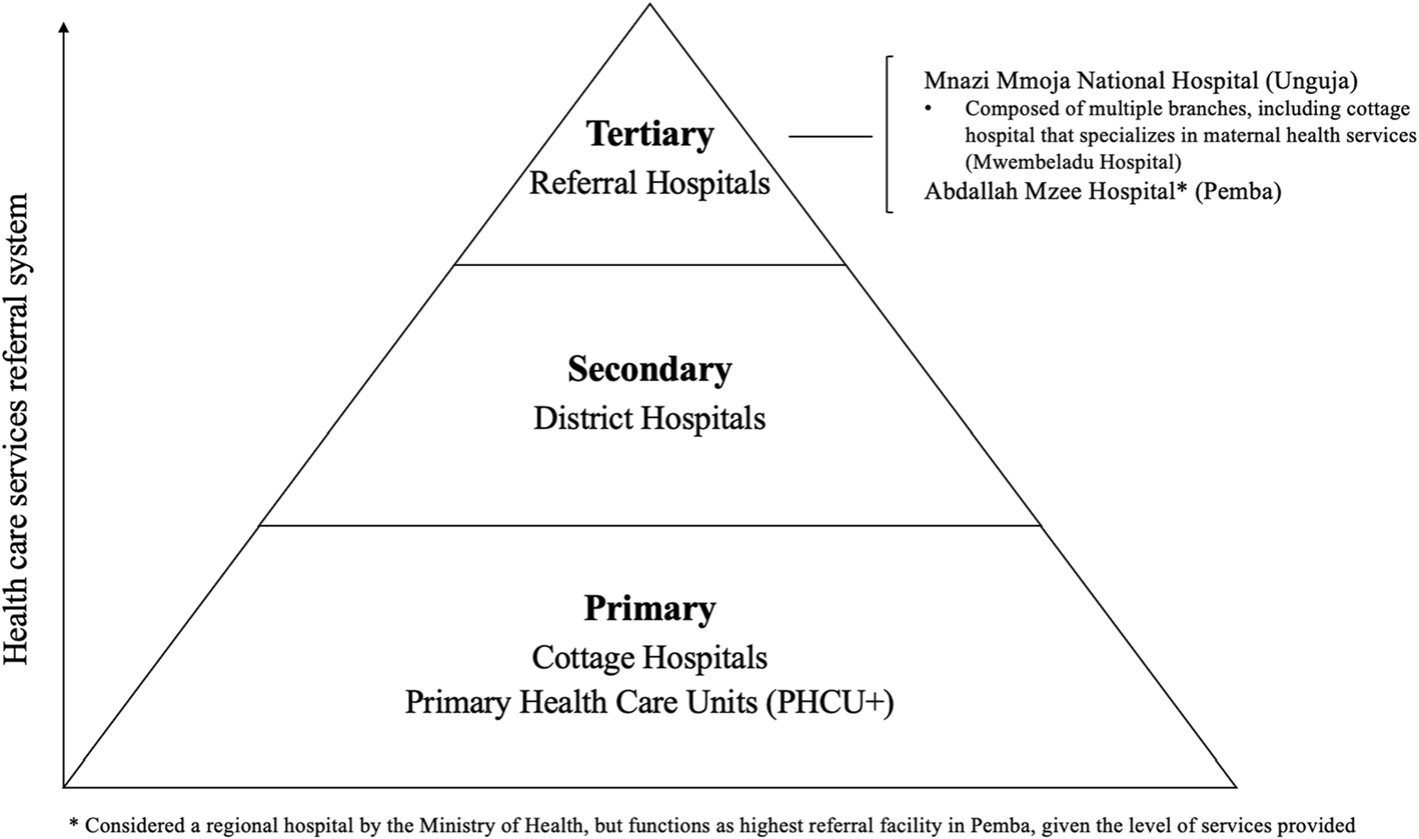
Health facilities in Zanzibar (Adapted from [37])

We were also interested in characteristics collected as part of the program after enrollment. In Zanzibar, levels of care and corresponding health facilities are segmented into three categories: a) primary level: health care units and centers, b) secondary level: district hospitals, c) tertiary level: hospitals that provide referral services (Fig. 2) [36]. For this analysis, the delivery facility types were categorized as: home/in community delivery, primary health care unit (PHCU +), cottage hospital, district hospital, referral hospital, other. A delivery facility location was recommended to women based on self-reported risk factors, including age, nulliparity or previous pregnancy complications, and pre-existing conditions. Patients who were at high risk were recommended by their CHW at the first visit to deliver at referral hospitals. Other variables include: delivery type (normal vaginal delivery (NVD), caesarean, other), and estimated cost from home to recommended health care facility for delivery (< 5,000 Tanzanian Shillings (TSH), 5,000–9,999 TSH, 10,000–14,999 TSH, 15,000–19,999 TSH, > 20,000 TSH). We estimated cost for delivery served as a proxy for distance, with higher cost indicating longer travel time from home to the recommended health facility. We also considered having at least one CHW postpartum phone consultation conducted over the phone (instead of a home visit), for women who were unable to receive a home visit because they indicated having plans to move away for delivery or were generally unable to be reached in person.

### Statistical analyses

#### Analysis 1. Completion of postpartum visits

We conducted Chi-squared tests to investigate the association between the receipt of all postpartum visits and each demographic characteristic. For the adjusted analysis, we used multivariate logistic regression model to assess the relationship between characteristics and receipt of all postpartum visits. We fit one model on information that was collected during the enrollment visit at baseline. We then fit a “full” model that included programmatic and delivery characteristics, while still adjusting for the baseline characteristics in the first model. These additional variables included delivery facility type, delivery type, estimated cost from home to recommended health care facility for delivery, and having at least one postpartum phone consultation.

#### Analysis 2. Time to first postpartum visit

We conducted two-sample t-tests and ANOVAs to investigate the association between mean time to first postpartum visit and each demographic characteristic. We used multivariate linear regression models to estimate whether there is significant differences in the mean time to first visit associated with changes in the level of covariates.

#### Additional considerations for regression models

For Analysis 1 and 2, we accounted for four potential biases in the regression models. First, the poverty assessment variables had 32.9% missingness, as these data were integrated into Logiak in March 2017 and were only collected at the 8-day postpartum visit. Moreover, they were not collected during this postpartum visit if the visit occurred by phone or if the first and only visit was the 8-day postpartum visit. To address this, we used multiple imputation by chained equations using the mice R package [38, 39]. We created 20 imputed datasets for our model fitting procedure. Second, it is possible that outcomes among women with the same CHW have correlated outcomes. To account for this potential clustering by CHW, we used generalized estimating equations with an exchangeable correlation structure (geepack R package) [40]. Third, for each analysis, we consider both a *baseline model*, containing only baseline characteristics, and a *full model*, containing all baseline and programmatic characteristics. The reason we fit both a baseline and full model is because the programmatic and delivery characteristics were collected after enrollment – commonly referred to as “mediators” or “intermediate variables” – and should not be adjusted for when trying to interpret the effects of preceding variables.

#### Analysis 3. Characteristics associated with LTFU

We also conducted a separate sub-analysis examining which characteristics associated with LTFU among the entire study population (*N* = 39,606). Chi-squared tests were used to investigate the relationship between demographic characteristics and LTFU status.

STATA IC/15.1 was used for data cleaning. R 4.0.3 was used for statistical analyses.

## Results

### Baseline characteristics

Table 1 provides information on sociodemographic, maternal health history, and health service utilization characteristics among all women in our study population (*N* = 33,914). The mean age of women enrolled in the Safer Deliveries Program was 27.4 years old. The majority of women were based in Mkoani (16.1%), Kaskazini A (15.4%), and Chake Chake (13.6%) districts. Over two-thirds (69.3%) of women had some form of formal education. When considering self-reported maternal health history characteristics of women, 14.5% and 2.7% had a previous spontaneous abortion and stillbirth, respectively. Almost all (96.0%) of women in the program are HIV-negative with 1.6% HIV-positive and 2.4% with unknown or unreported HIV status. The majority of women reported were either nulliparous (23%) or had previously given birth at most twice (33%). Five percent of women had previously given birth at least eight times. Nearly 12% of women reported having at least one previous pregnancy condition, while only 1.8% reported having at least one current pregnancy condition.

**Table 1:** Distribution of characteristics of women enrolled in the Safer Deliveries program (*N* = 33,914)

Characteristics	n	%	Missing (%)
**Age (years)**			
Mean[sd]	27.43[6.24]		
Median[25th, 75th]	27[23,31]		
< 20 years old	2,823	8.32	
20–29 years old	19,160	56.50	
30–39 years old	10,489	30.93	
> 40 years old	1,442	4.25	
**Education Level**			11,164 (32.92%)
No education	6,984	30.70	
Some Primary School	6,441	28.31	
Completed Primary School	3,937	17.31	
Some Secondary School	5,129	22.55	
Completed Secondary School	259	1.14	
**All Children Currently Living**			11,164 (32.92%)
Not all children living	2,446	10.75	
All children living	20,304	89.25	
**Electricity at Home**			11,164 (32.92%)
No electricity at home	14,810	65.10	
Electricity at home	7,940	34.90	
**Drinking Water Source**			11,164 (32.92%)
Surface water	187	0.82	
Tap pump outside	11,003	48.36	
Well	4,757	20.91	
Tap pump home	6,550	28.79	
Well home	184	0.81	
Other	69	0.30	
**Roof Material**			11,164 (32.92%)
Scrap corrugated iron	4,958	21.79	
Corrugated iron sheets	12,855	56.51	
Thatched	4,677	20.56	
Tiles / shingles	131	0.58	
Other	129	0.57	
**Floor Material**			11,164 (32.92%)
Dirt	6,543	28.76	
Plastic mat	241	1.06	
Concrete	15,683	68.94	
Tiles	199	0.87	
Other	84	0.37	
**District**			
Kaskazini B	2,679	7.90	
Kaskazini A	5,238	15.44	
Kati	3,117	9.19	
Maghribi	4,230	12.47	
Kusini	1,605	4.73	
Mkoani	5,456	16.09	
Wete	3,206	9.45	
Micheweni	3,762	11.09	
Chake Chake	4,621	13.63	
**HIV Status**			
Negative	32,557	96.00	
Positive	554	1.63	
Unknown or Unreported Status	803	2.37	
**Parity (births)**			
0	7,822	23.06	
1–2	11,205	33.04	
3–4	7,679	22.64	
5–7	5,513	16.26	
8 +	1,695	5.00	
**Abortion**			
Never had abortion	29,014	85.55	
Has had abortion	4,900	14.45	
**Stillbirth**			
Never had stillbirth	33,015	97.35	
Has had stillbirth	899	2.65	
**Previous Pregnancy Conditions**			69 (0.20%)
No previous conditions	29,929	88.43	
At least one previous condition	3,916	11.57	
**Current Pregnancy Conditions**			69 (0.20%)
No current conditions	33,234	98.19	
At least one current condition	611	1.81	
**Previous Delivery Location**			69 (0.20%)
No previous birth	7,822	23.11	
At home/in the community	7,093	20.96	
On the way to a health facility	292	0.86	
At health facility	18,638	55.07	
**Delivery Facility Type**			
Home/In community delivery	8,466	24.96	
Cottage hospital	12,811	37.77	
PHCU +	5,072	14.96	
Referral hospital	5,805	17.12	
District hospital	1,688	4.98	
Other	72	0.21	
**Type of Delivery**			8,436 (24.87)
NVD	24,122	94.68	
Caesarean	1,264	4.96	
Other	92	0.36	
**Cost From Home to Health Facility (Proxy for Distance)**			
< 5,000 TSH	12,046	35.52	
5,000 – 9,999 TSH	8,450	24.92	
10,000 – 14,999 TSH	4,267	12.58	
15,000 – 19,999 TSH	4,777	14.09	
> 20,000 TSH	4,374	12.90	
**Postpartum Phone Consultation**			
No postpartum phone consultation	28,564	84.22	
At least one postpartum phone consultation	5,350	15.78	

### Analysis 1. Completion of postpartum visits

Table 2 presents the bivariate analysis of whether receipt of the full set of visits differs according to sociodemographic and health characteristics. In the multivariate logistic regression model, delivery facility type was a significant predictor of receipt of the full postpartum intervention (Table 3), with women delivering at a PHCU + (OR = 1.75; *p* < 0.001; 95% Confidence Interval (95%CI): 1.35–2.27), cottage hospital (OR = 1.54; *P* = 0.002; 95%CI: 1.78–2.02) or home (OR = 1.37; *P* = 0.015; 95%CI: 1.06–1.76) being more likely to receive all three visits compared to those who delivered at a referral hospital. Other characteristics were negatively associated with the full intervention, such as parity, with women with eight or more previous births being less likely to receive all three visits compared to those with no previous birth (OR = 0.85; *P* = 0.014; 95%CI: 0.75–0.97). The estimated odds of the full intervention were also lower for women who received at least one postpartum phone consultation (OR = 0.77; *p* < 0.001; 95%CI: 0.69–0.87). Additionally, those who had an unknown or unreported HIV status were less likely to receive all three postpartum visits (OR = 0.64; *p* < 0.001; 95%CI: 0.53–0.78). Lastly, there were significant differences by districts, with those living in Kaskazini A being the most likely to receive all three visits compared to Kaskazini B (OR = 1.79; *P* = 0.03; 95%CI: 1.06–3.02). Wealth and education were not associated with receipt of the intervention. Additionally, other variables that were not statistically significant include: age, current pregnancy conditions, previous stillbirth, and cost of transportation to the recommended health facility.

**Table 2:** Characteristics by those who received all three postpartum visits (*N* = 33,914)

Characteristics	Total	Received all three visits		*p*-value
	**N**	**n**	**%**	
**Overall**	33,914	10,602	31.26	
**Age (years)**				0.259
< 20 years old	2,823	885	31.35	
20–29 years old	19,160	6,043	31.54	
30–39 years old	10,489	3,206	30.57	
> 40 years old	1,442	468	32.45	
**Education Level**				< 0.001
No education	6,984	2,276	32.59	
Some Primary School	6,441	2,124	32.97	
Completed Primary School	3,937	1,266	32.16	
Some Secondary School	5,129	1,965	38.31	
Completed Secondary School	259	98	37.84	
**All Children Currently Living**				0.022
Not all children living	2,446	882	36.06	
All children living	20,304	6,847	33.72	
**Electricity at Home**				< 0.001
No electricity at home	14,810	4,838	32.67	
Electricity at home	7,940	2,891	36.41	
**Drinking Water Source**				< 0.001
Surface water	187	49	26.20	
Tap pump outside	11,003	3,805	34.58	
Well	4,757	1,447	30.42	
Tap pump home	6,550	2,341	35.74	
Well home	184	72	39.13	
Other	69	15	21.74	
**Roof Material**				0.001
Scrap corrugated iron	4,958	1,646	33.20	
Corrugated iron sheets	12,855	4,441	34.55	
Thatched	4,677	1,574	33.65	
Tiles / shingles	131	45	34.35	
Other	129	23	17.83	
**Floor Material**				0.043
Dirt	6,543	2,136	32.65	
Plastic mat	241	92	38.17	
Concrete	15,683	5,412	34.51	
Tiles	199	63	31.66	
Other	84	26	30.95	
**District**				< 0.001
Kaskazini B	5,238	813	30.35	
Kaskazini A	2,679	1,974	37.69	
Kati	3,117	924	29.64	
Maghribi	4,230	1,225	28.96	
Kusini	1,605	524	32.65	
Mkoani	5,456	1,657	30.37	
Wete	3,206	849	26.48	
Micheweni	3,762	1,316	34.98	
Chake Chake	4,621	1,320	28.57	
**HIV Status**				< 0.001
Negative	32,557	10,315	31.68	
Positive	554	138	24.91	
Unknown or Unreported Status	803	149	18.56	
**Parity (births)**				< 0.001
0	7,822	2,623	33.53	
1–2	11,205	3,501	31.24	
3–4	7,679	2,322	30.24	
5–7	5,513	1,663	30.17	
8 +	1,695	493	29.09	
**Abortion**				
Never had abortion	29,014	9,088	31.32	0.564
Has had abortion	4,900	1,514	30.90	
**Stillbirth**				0.487
Never had stillbirth	33,015	10,331	31.29	
Has had stillbirth	899	271	30.90	
**Presence of Previous Pregnancy Conditions**				< 0.001
No previous conditions	29,929	9,457	31.60	
At least one previous condition	3,916	1,119	28.58	
**Presence of Current Pregnancy Conditions**				0.070
No current conditions	33,234	10,364	31.18	
At least one current condition	611	212	34.70	
**Previous Delivery Location**				< 0.001
No previous birth	7,822	2,623	33.53	
At home/in the community	7,093	2,065	29.11	
On the way to a health facility	292	81	27.74	
At health facility	18,638	5,807	31.16	
**Delivery Facility Type**				< 0.001
Home/In community delivery	8,466	2,539	29.99	
Cottage hospital	12,811	3,964	30.94	
PHCU +	5,072	1,870	36.87	
Referral hospital	5,805	1,713	29.51	
District hospital	1,688	485	28.73	
Other	72	31	43.06	
**Type of Delivery**				< 0.001
NVD	24,122	7,739	32.08	
Caesarean	1,264	297	23.50	
Other	92	41	44.57	
**Cost From Home to Health Facility (Proxy for Distance)**				0.003
< 5,000 TSH	12,046	3,636	30.18	
5,000 – 9,999 TSH	8,450	2,610	30.89	
10,000 – 14,999 TSH	4,267	1,373	32.18	
15,000 – 19,999 TSH	4,777	1,554	32.53	
> 20,000 TSH	4,374	1,429	32.67	
**Postpartum Phone Consultation**				< 0.001
No postpartum phone consultation	28,564	9,128	31.96	
At least one postpartum phone consultation	5,350	1,474	27.55

**Table 3:** Predictors of receipt of full postpartum intervention (*N* = 33,914)

	(A) **Baseline Model (Unadjusted associations)**			(B) **Full Model (Adjusted associations)**		
	**Odds Ratio**	**95% CI**	***P-value***	**Odds Ratio**	**95% CI**	***P***-value
**Intercept**	0.28	(0.16, 0.51)	< 0.001	0.20	(0.11, 0.39)	< 0.001
**Age (years)**						
< 20 years old	Reference					
20–29 years old	0.99	(0.91, 1.07)	0.825	1.00	(0.92, 1.08)	0.992
30–39 years old	0.97	(0.88, 1.07)	0.563	0.97	(0.88, 1.07)	0.56
> 40 years old	1.01	(0.88, 1.16)	0.928	1.01	(0.88, 1.16)	0.856
**Education Level**						
No education	Reference					
Some primary	1.00	(0.93, 1.08)	0.962	1.00	(0.93, 1.08)	0.934
Completed primary	0.95	(0.87, 1.04)	0.282	0.96	(0.88, 1.05)	0.392
Some secondary	1.04	(0.95, 1.14)	0.392	1.06	(0.97, 1.16)	0.197
Completed secondary	1.05	(0.85, 1.29)	0.660	1.08	(0.88, 1.32)	0.484
**All children currently living (yes)**	0.93	(0.84, 1.03)	0.166	0.92	(0.83, 1.02)	0.11
**Electricity at home (yes)**	1.06	(0.99, 1.14)	0.097	1.06	(0.99, 1.14)	0.087
**Drinking Water Source**						
Surface water	Reference					
Tap pump outside	1.30	(0.93, 1.81)	0.129	1.29	(0.92, 1.81)	0.135
Well	1.14	(0.80, 1.62)	0.470	1.15	(0.81, 1.63)	0.449
Tap pump home	1.35	(0.95, 1.90)	0.093	1.33	(0.94, 1.89)	0.106
Well home	1.39	(0.89, 2.18)	0.145	1.44	(0.92, 2.24)	0.108
Other	1.13	(0.68, 1.88)	0.634	1.12	(0.67, 1.86)	0.666
**Roof Material**						
Scrap corrugated iron	Reference					
Corrugated iron sheets	1.02	(0.94, 1.10)	0.630	1.01	(0.93, 1.10)	0.891
Thatched	0.98	(0.89, 1.08)	0.706	0.98	(0.88, 1.08)	0.622
Tiles / shingles	1.07	(0.72, 1.59)	0.734	1.06	(0.72, 1.57)	0.764
Other	0.75	(0.49, 1.15)	0.185	0.74	(0.48, 1.14)	0.177
**Floor Material**						
Dirt	Reference					
Plastic mat	1.08	(0.82, 1.43)	0.588	1.09	(0.83, 1.44)	0.543
Concrete	0.96	(0.89, 1.04)	0.318	0.97	(0.90, 1.05)	0.454
Tiles	0.85	(0.66, 1.10)	0.219	0.87	(0.67, 1.14)	0.312
Other	0.88	(0.53, 1.46)	0.614	0.88	(0.53, 1.45)	0.608
**District**						
Kaskazini B	Reference					
Kaskazini A	1.79	(1.06, 3.02)	0.030	1.74	(1.04, 2.92)	0.034
Kati	1.05	(0.60, 1.85)	0.868	1.17	(0.66, 2.09)	0.584
Maghribi	1.33	(0.76, 2.32)	0.319	1.31	(0.76, 2.28)	0.337
Kusini	1.51	(0.81, 2.82)	0.195	1.31	(0.70, 2.44)	0.396
Mkoani	1.25	(0.72, 2.16)	0.424	1.38	(0.80, 2.40)	0.251
Wete	1.21	(0.68, 2.16)	0.520	1.08	(0.61, 1.90)	0.80
Micheweni	1.57	(0.90, 2.73)	0.114	1.43	(0.82, 2.49)	0.206
Chake Chake	1.09	(0.63, 1.88)	0.759	1.12	(0.65, 1.92)	0.69
**HIV Status**						
Negative	Reference					
Positive	1.02	(0.86, 1.21)	0.826	1.02	(0.86, 1.21)	0.851
Unknown or Unreported	0.64	(0.53, 0.78)	< 0.001	0.71	(0.59, 0.86)	< 0.001
**Parity (births)**						
0	Reference					
1–2	0.94	(0.88, 1.00)	0.035	0.92	(0.86, 0.98)	0.009
3–4	0.91	(0.84, 0.98)	0.017	0.89	(0.82, 0.96)	0.004
5–7	0.91	(0.84, 0.99)	0.031	0.90	(0.82, 0.98)	0.014
8 +	0.85	(0.75, 0.97)	0.014	0.84	(0.74, 0.96)	0.008
**Previous abortion (yes)**	1.07	(1.01, 1.14)	0.017	1.08	(1.01, 1.14)	0.015
**Previous stillbirth (yes)**	1.00	(0.88, 1.15)	0.953	1.01	(0.88, 1.15)	0.921
**Current pregnancy conditions present (yes)**	1.07	(0.90, 1.26)	0.460	1.06	(0.90, 1.25)	0.491
**Delivery Facility Type**						
Referral hospital				Reference		
Home/In community delivery				1.37	(1.06, 1.76)	0.015
Cottage hospital				1.54	(1.17, 2.02)	0.002
PHCU +				1.75	(1.35, 2.27)	< 0.001
District hospital				1.29	(0.99, 1.68)	0.064
Delivery Facility Type: Other				2.26	(1.38, 3.72)	0.001
**Cost From Home to Health Facility (Proxy for Distance)**						
< 5,000 TSH				Reference		
5,000—9,999 TSH				0.96	(0.85, 1.08)	0.509
10,000—14,999 TSH				1.02	(0.90, 1.16)	0.715
15,000- 19,999 TSH				1.06	(0.95, 1.20)	0.311
> 20,000 TSH				1.07	(0.95, 1.20)	0.248
**Receipt of at least one postpartum phone consultation (yes)**				0.77	(0.69, 0.87)	< 0.001

#### Analysis 2. Time to first postpartum visit

**Fig. 3:**
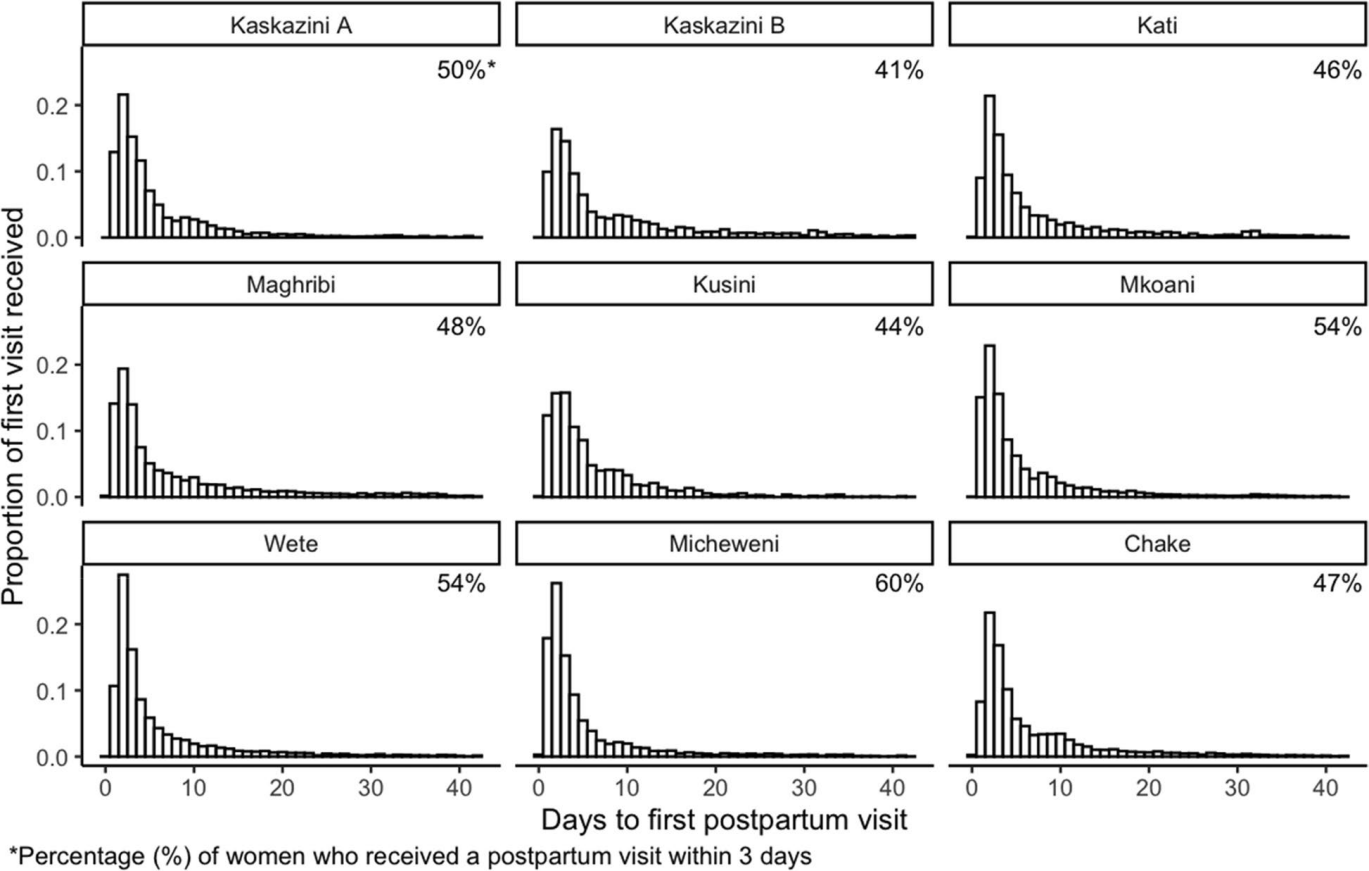
Distribution of women by number of days to the first postpartum visit by district (*N* = 33,914)

Figure 3 provides the distribution of women by the number of days to the first postpartum visit within the 42-day postpartum period, stratified by district. Across all districts, at least 64% of women received their first postpartum visit within the first week post-delivery. Districts with the most women receiving their first postpartum visit within three days of delivery include Micheweni (60%), Wete (54%), and Mkoani (54%) (Fig. 3).

Table 4 describes the mean time to the first postpartum visit by each unadjusted variable. When examining the regression analysis output for the adjusted model (Table 5), similar cross-sectional associations to the previous model (Table 3) were observed—women who delivered at a cottage hospital (estimated mean difference of -0.85 days, *P* = 0.009; 95%CI: -1.49–-0.22) or PHCU + (estimated mean difference of -1.28 days, *p* < 0.001; 95%CI: -1.92–-0.64) had shorter average times to first postpartum visit than women who delivered at a referral hospital. Delivering at home was also associated with a shorter waiting period between birth and the first postpartum visit (estimated mean difference of -0.90 days; *P* = 0.005; 95%CI: -1.52–-0.28). All districts had shorter times to first postpartum visit than Kaskazini B, with Mkoani (estimated mean difference of -2.99 days; *p* < 0.001; 95%CI: -4.79–-1.20) and Micheweni (estimated mean difference of -3.53 days; *p* < 0.001; 95%CI: -5.44–-1.62) having their first visits at least 3 days earlier on average. However, women who received at least one postpartum phone consultation, on average, had their first visit almost a full day later than those who did not have a phone consultation (estimated mean difference of 0.83 days; *p* < 0.001; 95%CI: 0.43–1.23). Moreover, women with an unknown or unreported HIV status, on average, experienced a 1.8-day delay to their first visit than those who were HIV negative (estimated mean difference of 1.81 days; *p* < 0.001; 95%CI: 1.03–2.59). The following variables were not statistically significant in the model: age, poverty assessment indicators (i.e., education level, electricity access, drinking water access, all children currently living, roof material, and floor), parity, current pregnancy conditions, previous abortion, previous stillbirth, and cost of transportation to the recommended health facility.

**Table 4:** Characteristics by time to first postpartum visit (*N* = 33,914)

Characteristics	Time to first postpartum visit		*p*-value
	**Mean[sd]**	**Median** **[25th, 75th]**	
**Age (years)**			0.343
< 20 years old	6.61[7.41]	4[2, 8]	
20–29 years old	6.66[7.66]	3[2, 8]	
30–39 years old	6.59[7.50]	4[2, 8]	
> 40 years old	6.43[7.23]	3[2, 8]	
**Education Level**			0.536
No education	6.10[7.41]	3[2, 6]	
Some Primary School	6.46[7.58]	3[2, 7]	
Completed Primary School	6.76[7.83]	4[2, 8]	
Some Secondary School	5.93[7.22]	3[2, 6]	
Completed Secondary School	5.78[7.31]	3[2, 7]	
**All Children Currently Living**			0.0190
Not all children living	5.95[7.37]	2[2, 6]	
All children living	6.32[7.51]	3[2, 7]	
**Electricity at Home**			0.763
No electricity at home	6.29[7.54]	3[2, 7]	
Electricity at home	6.26[7.42]	3[2, 7]	
**Drinking Water Source**			0.674
Surface water	6.14[6.91]	3[2, 8]	
Tap pump outside	6.26[7.46]	3[2, 7]	
Well	6.52[7.87]	3[2, 7]	
Tap pump home	6.12[7.26]	3[2, 7]	
Well home	5.98[7.68]	3[2, 6]	
Other	8.59[9.40]	5[3, 10]	
**Roof Material**			0.006
Scrap corrugated iron	6.30[7.57]	3[2, 7]	
Corrugated iron sheets	6.41[7.53]	3[2, 7]	
Thatched	5.92[7.35]	3[2,76]	
Tiles / shingles	5.98[6.81]	4[2, 6]	
Other	5.50[6.42]	3[2, 6]	
**Floor Material**			0.001
Dirt	6.05[7.33]	3[2, 6]	
Plastic mat	5.12[7.07]	3[1, 6]	
Concrete	6.38[7.56]	3[2, 7]	
Tiles	6.57[7.26]	4[2, 8]	
Other	7.04[8.85]	3[1, 11]	
**District**			< 0.001
Kaskazini B	8.42[8.94]	4[2, 11]	
Kaskazini A	5.95[6.62]	4[2, 7]	
Kati	7.51[8.46]	4[2, 9]	
Maghribi	7.75[8.84]	4[2, 10]	
Kusini	6.34[6.37]	4[2, 8]	
Mkoani	6.03[7.04]	3[2, 7]	
Wete	7.58[7.20]	3[2, 7]	
Micheweni	6.15[6.78]	3[2, 6]	
Chake Chake	5.41[7.35]	4[2, 9]	
**HIV Status**			< 0.001
Negative	6.57[7.52]	3[2, 8]	
Positive	6.60[8.32]	3[2, 7]	
Unknown or Unreported Status	8.99[9.05]	5[3,11.5]	
**Parity (births)**			0.449
0	6.54[7.62]	3[2, 8]	
1–2	6.73[7.68]	4[2, 8]	
3–4	6.71[7.57]	4[2, 8]	
5–7	6.42[7.38]	3[2, 8]	
8 +	6.56[7.45]	4[2, 8]	
**Abortion**			0.679
Never had abortion	6.62[7.57]	3[2, 8]	
Has had abortion	6.66[7.65]	4[2, 8]	
**Stillbirth**			0.314
Never had stillbirth	6.63[7.59]	4[2, 8]	
Has had stillbirth	6.38[7.34]	3[2, 8]	
**Previous Pregnancy Conditions**			0.145
No previous conditions	6.65[7.61]	4[2, 8]	
At least one previous condition	6.46[7.35]	4[2, 8]	
**Current Pregnancy Conditions**			0.747
No current conditions	6.63[7.57]	4[2, 9]	
At least one current condition	6.53[7.85]	3[2, 8]	
**Previous Delivery Location**			0.001
No previous birth	6.54[7.62]	3[2, 8]	
At home/in the community	6.34[7.28]	3[2, 8]	
On the way to a health facility	6.62[7.68]	4[2, 8]	
At health facility	6.77[7.67]	4[2, 8]	
**Delivery Facility Type**			< 0.001
Home/In community delivery	6.42[7.54]	3[2, 8]	
Cottage hospital	6.59[7.30]	4[2, 8]	
PHCU +	5.95[7.17]	3[2, 7]	
Referral hospital	7.71[8.48]	4[2, 10]	
District hospital	6.17[7.38]	3[2, 7]	
Other	7.06[8.13]	3[2,9.25]	
**Type of Delivery**			0.182
NVD	6.66[7.59]	4[2, 8]	
Caesarean	7.33[7.65]	5[2, 9]	
Other	4.46[4.98]	3[2, 4]	
**Cost From Home to Health Facility (Proxy for Distance)**			0.0618
< 5,000 TSH	6.68[7.67]	4[2, 8]	
5,000 – 9,999 TSH	6.82[7.93]	4[2, 8]	
10,000 – 14,999 TSH	6.32[7.17]	3[2, 8]	
15,000 – 19,999 TSH	6.28[7.04]	4[2, 8]	
> 20,000 TSH	6.74[7.60]	3[2, 9]	
**Postpartum Phone Consultation**			< 0.001
No postpartum phone consultation	6.49[7.48]	3[2, 8]	
At least one postpartum phone consultation	7.35[8.04]	4[2, 9]

**Table 5:** Predictors of time to first postpartum visit (*N* = 33,914)

	(A) **Baseline Model (Unadjusted associations)**			(B) **Full Model (Adjusted associations)**		
	**Mean Estimate**	**95% CI**	***P*-value**	**Mean Estimate**	**95% CI**	***P*-value**
**Intercept**	9.23	(7.24, 11.21)	< 0.001	9.69	(7.61, 11.77)	< 0.001
**Age (years)**						
< 20 years old	Reference					
20–29 years old	0.11	(-0.19, 0.41)	0.472	0.11	(-0.19, 0.41)	0.484
30–39 years old	0.06	(-0.31, 0.42)	0.761	0.07	(-0.30, 0.43)	0.723
> 40 years old	-0.15	(-0.64, 0.34)	0.553	-0.16	(-0.65, 0.32)	0.513
**Education Level**						
No education	Reference					
Some primary	-0.02	(-0.29, 0.24)	0.864	-0.05	(-0.31, 0.22)	0.737
Completed primary	0.38	(0.06, 0.70)	0.020	0.34	(0.02, 0.66)	0.038
Some secondary	-0.14	(-0.46, 0.18)	0.391	-0.21	(-0.53, 0.10)	0.184
Completed secondary	-0.02	(-0.84, 0.81)	0.967	-0.11	(-0.93, 0.71)	0.792
**All children currently living (yes)**	0.00	(-0.32, 0.32)	0.999	0.029	(-0.29, 0.34)	0.859
**Electricity at home (yes)**	-0.10	(-0.34, 0.15)	0.439	-0.11	(-0.34, 0.13)	0.391
**Drinking Water Source**						
Surface water	Reference					
Tap pump outside	0.03	(-0.98, 1.04)	0.953	0.06	(-0.95, 1.06)	0.913
Well	0.34	(-0.67, 1.35)	0.509	0.33	(-0.67, 1.34)	0.515
Tap pump home	-0.19	(-1.13, 0.91)	0.835	-0.07	(-1.08, 0.94)	0.891
Well home	0.13	(-1.24, 1.49)	0.855	0.06	(-1.28, 1.40)	0.931
Other	0.48	(-1.41, 2.36)	0.622	0.49	(-1.39, 2.36)	0.612
**Roof Material**						
Scrap corrugated iron	Reference					
Corrugated iron sheets	-0.15	(-0.42, 0.11)	0.256	-0.11	(-0.37, 0.15)	0.412
Thatched	-0.01	(-0.32, 0.30)	0.951	0.02	(-0.29, 0.33)	0.899
Tiles / shingles	-0.48	(-1.84, 0.89)	0.492	-0.44	(-1.78, 0.89)	0.515
Other	-0.92	(-1.94, 0.11)	0.080	-0.90	(-1.91, 0.12)	0.085
**Floor Material**						
Dirt	Reference					
Plastic mat	-1.42	(-2.35, -0.48)	0.003	-1.45	(-2.37, -0.53)	0.002
Concrete	0.07	(-0.21, 0.34)	0.633	0.034	(-0.24, 0.31)	0.808
Tiles	0.10	(-0.91, 1.10)	0.853	0.005	(-1.0, 1.01)	0.992
Other	1.12	(-0.80, 3.03)	0.254	1.11	(-0.80, 3.02)	0.254
**District**						
Kaskazini B	Reference					
Kaskazini A	-2.90	(-4.67, -1.13)	0.001	-2.82	(-4.60, -1.03)	0.002
Kati	-1.59	(-3.58, 0.40)	0.117	-1.70	(-3.70, 0.30)	0.096
Maghribi	-1.28	(-3.20, 0.63)	0.189	-1.38	(-3.31, 0.55)	0.161
Kusini	-2.83	(-4.81, -0.85)	0.005	-2.88	(-4.88, -0.88)	0.005
Mkoani	-2.99	(-4.79, -1.20)	0.001	-3.15	(-4.95, -1.35)	< 0.001
Wete	-2.89	(-4.92, -0.85)	0.005	-2.84	(-4.89, -0.79)	0.007
Micheweni	-3.53	(-5.44, -1.62)	< 0.001	-3.33	(-5.26, -1.40)	< 0.001
Chake Chake	-1.90	(-3.75, -0.05)	0.045	-1.84	(-3.72, 0.04)	0.054
**HIV Status**						
Negative	Reference					
Positive	0.11	(-0.72, 0.94)	0.791	0.143	(-0.68, 0.97)	0.733
Unknown or Unreported	1.81	(1.03, 2.59)	< 0.001	1.51	(0.75, 2.27)	< 0.001
**Parity (births)**						
0	Reference					
1–2	0.03	(-0.20, 0.26)	0.822	0.15	(-0.09, 0.39)	0.215
3–4	0.06	(-0.22, 0.34)	0.662	0.20	(-0.08, 0.49)	0.165
5–7	-0.04	(-0.35, 0.28)	0.818	0.11	(-0.21, 0.44)	0.504
8 +	0.20	(-0.24, 0.64)	0.381	0.36	(-0.09, 0.80)	0.114
**Previous abortion (yes)**	-0.13	(-0.34, 0.08)	0.237	-0.12	(-0.33, 0.08)	0.242
**Previous stillbirth (yes)**	-0.36	(-0.80, 0.08)	0.105	-0.40	(-0.83, 0.04)	0.074
**Current pregnancy conditions present (yes)**	0.11	(-0.40, 0.63)	0.666	0.12	(-0.40, 0.64)	0.659
**Delivery Facility Type**						
Referral hospital				Reference		
Home/In community delivery				-0.90	(-1.52, -0.28)	0.005
Cottage hospital				-0.85	(-1.49, -0.22)	0.009
PHCU +				-1.28	(-1.92, -0.64)	< 0.001
District hospital				-0.43	(-1.12, 0.27)	0.233
Delivery Facility Type: Other				-1.05	(-2.56, 0.45)	0.171
**Cost From Home to Health Facility (Proxy for Distance)**						
< 5,000 TSH				Reference		
5,000—9,999 TSH				0.12	(-0.23, 0.47)	0.491
10,000—14,999 TSH				0.07	(-0.34, 0.48)	0.748
15,000- 19,999 TSH				0.14	(-0.21, 0.48)	0.438
> 20,000 TSH				0.40	(0.028, 0.77)	0.035
**Receipt of at least one postpartum phone consultation (yes)**				0.83	(0.43 1.23)	< 0.001

#### Analysis 3. Characteristics associated with LTFU

Table 6 examines characteristics prior to the postpartum follow-up period disaggregated by LTFU within the larger study population (*N* = 39,606). The following characteristics were associated with LTFU (*P* < 0.05): age, district, HIV status, parity, abortion, previous delivery location, and cost of transportation to recommended health facility. Younger women (16.9% under 20 years of age vs. 11.8% over 40 years of age; *p* < 0.001) and those with lower parity (17.6% with no prior births vs. 9.1% with 8 + prior births; *p* < 0.001) were more likely to be LTFU. Likewise, 23.4% of women from Kaskazini A and 24.8% of those from Maghribi were LTFU (*p* < 0.001). Sixty-four percent of women with an unknown or unreported HIV status were LTFU compared to only 11.3% of those that were HIV-negative (*p* < 0.001).

**Table 6:** Characteristics disaggregated by LTFU (*N* = 39,606)

Characteristics	Total	Loss to follow-up		*p*-value
	**N**	**n**	**%**	
**Overall**	36,606	5,692	14.37	
**Age (years)**				< 0.001
< 20 years old	3,396	573	16.87	
20–29 years old	22,550	3,390	15.03	
30–39 years old	12,025	1,536	12.77	
> 40 years old	1,635	193	11.80	
**District**				< 0.001
Kaskazini B	5,826	820	23.44	
Kaskazini A	3,499	588	10.09	
Kati	3,805	688	18.08	
Maghribi	5,626	1,396	24.81	
Kusini	1,949	344	17.65	
Mkoani	5,999	543	9.05	
Wete	3,545	339	9.56	
Micheweni	4,253	491	11.54	
Chake Chake	5,104	483	9.46	
**HIV Status**				< 0.001
Negative	36,713	4,156	11.32	
Positive	652	98	15.03	
Unknown or Unreported Status	2,241	1,438	64.17	
**Parity (births)**				< 0.001
0	9,493	1,671	17.60	
1–2	13,258	2,053	15.48	
3–4	8,845	1,166	13.18	
5–7	6,145	632	10.28	
8 +	1,865	170	9.12	
**Abortion**				0.011
Never had abortion	33,957	4,943	14.56	
Has had abortion	5,649	749	13.26	
**Stillbirth**				0.576
Never had stillbirth	38,564	5,549	14.39	
Has had stillbirth	1,042	143	13.72	
**Presence of Previous Pregnancy Conditions**				0.406
No previous conditions	34,966	5,037	14.41	
At least one previous condition	4,550	634	13.93	
**Presence of Current Pregnancy Conditions**				0.657
No current conditions	38,808	5,574	14.36	
At least one current condition	708	97	13.70	
**Previous Delivery Location**				< 0.001
No previous birth	9,493	1,671	17.60	
At home/in the community	7,928	835	10.53	
On the way to a health facility	343	51	14.87	
At health facility	21,752	3,114	14.32	
**Cost From Home to Health Facility (Proxy for Distance)**				< 0.001
< 5,000 TSH	14,187	2,141	15.09	
5,000 – 9,999 TSH	9,856	1,406	14.27	
10,000 – 14,999 TSH	5,026	759	15.10	
15,000 – 19,999 TSH	5,465	688	12.59	
> 20,000 TSH	5,061	687	15.71	

## Discussion

Within the Safer Deliveries program, we found that greater parity was significantly associated with a decreased likelihood of receiving all postpartum home visits from the CHW. Previous studies have indicated a similar relationship between parity and utilization of MNCH services access. A study by Mohan et al. (2017) suggested that higher parity was consistently associated with dropout from care continuum among women in Tanzania[23]. Moreover, other studies indicated that women with higher parity were more likely to delay PNC and have fewer PNC visits [41–44]. This may be because women who have had previous pregnancies rely on their past experiences and may not see the benefit in continuing with postpartum visits. Another explanation is that CHWs may perceive these women as being more knowledgeable and therefore, may not prioritize visiting them as often.

Having a delivery within a health facility (e.g., cottage hospital, PHCU + , district hospital) as opposed to a referral hospital increased likelihood of receiving all three visits. The Safer Deliveries utilized a set of risk criteria based on Ministry of Health guidance to recommend delivery locations, where high risk women were recommended to deliver at a referral hospital. Further, if a pregnancy cannot be managed at a lower facility, women may be referred to a different facility. As there is only one referral hospital on each island and in Unguja it is outside of the Safer Deliveries implementation districts, women who go to a referral hospital may travel to and stay in town near the facility before and/or after the delivery for monitoring. These women may even relocate to be with family for the duration of the postpartum period, sometimes without the CHW’s awareness. This could cause a delay in the CHW postpartum visit as CHWs work to locate and get in touch with their clients. Thus, there may be delays in receipt of CHW home visits for women who require the highest referral facility, who may want to remain near the hospital for monitoring before returning home. There may be delays in receipt of CHW home visits for women who require the highest referral facility, who may want to remain near the hospital for monitoring before returning home. Additionally, maternal complications, which may be handled at more equipped higher-level facilities, are also associated with longer hospital stays [45]. Women who deliver at home received their first visit earlier than those who delivered at a referral hospital as it may be easier for CHWs to immediately reach women who remain within the catchment areas the CHWs serve. CHWs are also trained to prioritize postpartum visits to women who delivered at home in order to check on the woman and baby’s health status and encourage them to visit a facility if they have not yet done so.

Within districts, women from Kaskazini A were more likely to receive all postpartum home visits compared to women from Kaskazini B. Further, women from every district received their first postpartum visit earlier than women from Kaskazini B. On average, women from Mkoani and Micheweni had their first postpartum visit at least 3 days before women from Kaskazini B. Although the program was first implemented in Kaskazini B, this district has poor facility coverage, lacking access to cottage and district hospitals [46]. This may mean that women need to travel further for delivery for an adequate facility—some women travel to Kivunge, a cottage hospital in Kaskazini A that is closer. Since women may move out of Kaskazini B for delivery, CHWs may find it more difficult to contact these women, which leads to delays in timely receipt of the intervention. Additionally, among districts in Zanzibar, Kaskazini B generally performs poorly in other indicators, such as immunization and ANC services [47], with a lower percentage of women receiving facility PNC checkups [15]. This suggests a need to focus resources and programmatic efforts on Kaskazini B to ensure sufficient support for women and newborns during the postpartum period.

Women with an unknown or unreported HIV status were less likely to receive all postpartum home visits and on average, experienced a 1.5-day delay to their first visit than those who were HIV negative. They were also disproportionately LTFU (64.2% vs. 11.41% with known HIV status). There may be a number of reasons for these findings. If a woman does not know her HIV status, CHWs will encourage her to get tested and will subsequently update information during a follow-up visit. However, if there is no additional pregnancy or postpartum visits for this client, there is less opportunity for this information to be collected. This may explain the higher proportion of women with unknown or unreported HIV status among LTFU, indicating a potential measurement error (included in limitations). Women who engage less with healthcare may be more likely to have an unknown or unreported HIV status and may also have a lower likelihood to receive all visits. A study by Kalichman and Rompa observed that lower health literacy was associated with poorer knowledge of one's HIV-related health status [48], although another study suggested it is not a barrier to HIV testing when recommended by a health professional [49]. Women may also not want to disclose or find or find out their HIV status, resulting in program attrition. This is consistent with previous studies in sub-Saharan Africa, which have indicated that pregnant women avoid health clinics if they fear being HIV tested or having their test results involuntarily disclosed [50, 51]. Specifically, studies in Kenya, Malawi and Uganda indicated that women of unknown HIV status avoid facility delivery to avoid potential association with HIV in the community, highlighting the impact of stigma [52–54]. Moreover, in-depth qualitative data in Kenya revealed that these women were likely to be targets of stigma and discriminatory practices during labor and delivery and were not receiving needed counseling services [55]. However, it is important to note that the prevalence of HIV in Zanzibar stands at less than 1%, which is lower than neighboring countries and those referenced in these studies [56]. Within the Safer Deliveries program, low health literacy or fear of discrimination and stigma may impede overall maternal health-seeking behaviors, underscoring the importance of integrating HIV-specific trainings among CHWs and the communities in which they serve.

We also found that on average, women who had a postpartum phone consultation were less likely to receive the entire postpartum intervention and received their first postpartum visit almost a whole day after women who did not receive a phone consultation. The intervention is a home-based visit; however, the phone option is available when CHWs are unable to reach mothers (e.g., woman moved away for delivery, woman lives very far from the CHW, CHW is unable to make it to residence). In general, CHWs are not informed by the health facility when a woman delivers and are dependent on mothers calling to notify them or by word of mouth. Therefore, when women are no longer within the same village as the CHW (and may not return for some time during the postpartum period), it makes it more difficult for the CHW to determine if a woman delivered, and therefore, more difficult for the woman to receive a timely home visit. If a CHW is unable to deliver the intended intervention in-person, they may initiate a phone consultation instead to avoid further delays.

Most characteristics in the models yielded no differences in receipt of the postpartum intervention, once adjusting for other baseline variables. In particular, this is the case for poverty assessment variables, which did not indicate potential inequities in programmatic outcomes. When examining these results against current literature, there appears to be similar findings on the effect of SES on equity. One systematic review focused on the reduction of socioeconomic inequities by coverage of CHW-facilitated interventions, including home visits. Findings revealed that CHW interventions improved equity in the distribution of maternal and newborn health outcomes between wealth quintiles or education level [57]. Moreover, Quayyum et al. (2013) found that the CHW intervention significantly increased facility PNC within 48 h, with equitable improvements across wealth [58]. Therefore, the lack of differences in outcomes across wealth and education levels in this study may suggest that the Safer Deliveries program is effectively reaching women and improving utilization of maternal health care regardless of SES.

This study has several limitations that should be considered. For our final study population, we excluded 5,692 women (14.4%) that are LTFU. These women were more likely to be younger and have an unknown or unreported HIV status, younger women may move to their family’s home for delivery. First-time mothers may be more likely to do this because they do not have children at home and there is less urgency to return. An explanation for LTFU among those with unknown or unreported HIV status was examined above. As a result, a separate analysis was conducted with LTFU. Although these women were not included in the main analysis because the LTFU occurred prior to delivery, this may reflect a gap in programming as these women were less likely to receive the program as intended. Second, since the study only considered women that were enrolled in the Safer Deliveries program, there may be characteristics that women in this population share that is not generalizable to other populations, such as health-seeking behavior that influenced program enrollment and cultural differences that impact postnatal care beliefs and practices. Moreover, it is important to note information on previous pregnancies and associated complications were self-reported. Although CHWs are trained to discuss these elements in detail with women, it is possible that biases from maternal recall and lack of understanding of specific conditions were introduced in the data. Finally, the data were missing poverty assessment variable information for 11,164 women. Collection of this data did not start until March 2017 and were only collected at the 8-day postpartum visit. Since both outcomes are related to the postpartum visits themselves, the variables were missing more often for women who did not have a postpartum visit. This necessitated using multiple imputation for missing data to improve validity of results, however, the validity of this procedure is predicated under the assumption that the data are missing at random conditional on the observed demographic characteristics collected by the program.

## Conclusions

Addressing inequities in access to maternal healthcare is vital to achieving international and national-level development objectives and reducing overall maternal and neonatal mortality and morbidity. The Safer Deliveries program promotes access to and utilization of health services and improves the overall quality of maternal and neonatal health care, regardless of one’s socioeconomic status. Beyond this, the study provides a better understanding of which groups of women are differentially served and enables future programs to develop targeted approaches to reach these specific groups of women and sustain health equity. These groups include women with higher parity, those who deliver at referral hospitals, those who reside in areas with limited resources, and women with an unknown or unreported HIV status. We suggest incorporating quality improvement measures to monitor and understand performance gaps as they relate to equity as necessary to improving overall programming. Additionally, more education for CHWs around the importance of postpartum visits may potentially shorten time to first visits.

## Data Availability

The data that support the findings of this study are available from D-tree International, but restrictions apply to the availability of these data, which were used under license for the current study, and therefore are not publicly available. Data are however available from the authors upon reasonable request and with permission of D-tree International and the Zanzibar Ministry of Health. To request data from this study, please contact Dr. Isabel Fulcher at ifulcher@d-tree.org.

## Abbreviations

CHW: Community health worker
LTFU: Lost to follow up
MMR: Maternal mortality ratio
NVD: Normal vaginal delivery
OR: Odds ratio
PHCU+: Primary health care unit
PNC: Postnatal care
SES: Socioeconomic status
TDHS: Tanzania demographic health survey
TSH: Tanzanian shillings
WHO: World health organization
